# TEAD3 + high-risk melanoma cells crosstalk with GAS6 + macrophages via the GAS6-TYRO3 ligand-receptor axis to modulate propionate metabolism and drive melanoma progression

**DOI:** 10.1186/s13046-025-03542-0

**Published:** 2025-10-01

**Authors:** Yongjin Fang, Xiaofan Xu, Rihui Lu, Ye Huang, Xinshen Dai, Pucheng Huang, Xuefeng Fu, Pan Zhuge

**Affiliations:** 1 Department of Otolaryngology Head and Neck Surgery, Affiliated Jinhua Hospital, Zhejiang University School of Medicine, No. 365, East renmin Rd, wucheng District, Jinhua, 321000 Zhejiang Province China; 2 Department of Health Technology and Informatics, The Hong Kong Polytechnic University, Hong Kong SAR, China; 3 Department of Dermatology, Affiliated Jinhua Hospital, Zhejiang University School of Medicine, No. 365, East renmin Rd, wucheng District, Jinhua, 321000 Zhejiang Province China

**Keywords:** Melanoma heterogeneity, TEAD3, GAS6-TYRO3 axis, Propionate metabolism, Immunotherapy resistance

## Abstract

**Background:**

Melanoma, a highly heterogeneous malignancy, remains refractory to conventional therapies due to poorly defined molecular and metabolic drivers. Short-chain fatty acid (SCFA) metabolism influences tumor progression, yet its role in melanoma subtypes and clinical outcomes is unclear. This study aims to delineate melanoma subgroups driven by SCFA metabolic dysregulation and identify mechanisms underlying their aggressiveness.

**Methods:**

Using non-negative matrix factorization (NMF), we clustered 468 TCGA melanoma samples into six subgroups based on SCFA-related gene sets (GO:0019745, GO:0019746, GO:0006085). Survival, differential expression, and pathway analyses were performed to characterize high-risk subgroups. Key drivers were validated via CRISPR/Cas9, siRNA knockdown, and immunohistochemistry. Single-cell RNA-seq (GSE215120) and spatial transcriptomics elucidated tumor-microenvironment crosstalk. Metabolic profiling, Seahorse assays, and myeloid-specific GAS6 knockout models were employed to dissect mechanisms.

**Results:**

NMF clustering revealing a high-risk subtype (Group 6) with dysregulated short-chain fatty acid (SCFA) metabolism and poor survival. Group 6 exhibited upregulation of GLTP and RAPGEFL1, enrichment in melanogenesis, Hippo signaling, and skin/lipid metabolism pathways. Through integrative analysis, TEAD3 emerged as a key risk driver, with high expression correlating with poor prognosis. Functional validation demonstrated that TEAD3 knockout suppressed melanoma proliferation, migration, and epithelial-mesenchymal transition (EMT) in vitro and in vivo. Single-cell RNA sequencing of acral melanoma revealed TEAD3-enriched tumor cells interacting with M2 macrophages via the GAS6-TYRO3 axis. Mechanistically, GAS6 + macrophages exhibited hypermetabolic phenotypes (elevated glycolysis/OXPHOS) that fueled GAS6 secretion. GAS6-TYRO3 signaling in TEAD3 + cells drove tumor aggressiveness by rewiring propionate metabolism, inducing methylmalonic acid accumulation via Mmut upregulation. Targeting this axis in myeloid-specific GAS6 knockout mice enhanced anti-PD-1 efficacy, boosting CD8 + T cell infiltration and survival.

**Conclusion:**

We define a TEAD3-driven melanoma subtype reliant on SCFA metabolic reprogramming and M2 macrophage crosstalk. The GAS6-TYRO3 axis and Mmut-mediated methylmalonic acid accumulation represent actionable targets. Combining myeloid-GAS6 ablation with immune checkpoint blockade overcomes therapy resistance, offering a precision strategy for high-risk melanoma.

**Supplementary Information:**

The online version contains supplementary material available at 10.1186/s13046-025-03542-0.

## Background

Acral melanoma, a highly aggressive skin cancer originating from melanocytes, has seen a rising global incidence over the past decades [1]. Despite advancements in targeted therapies and immune checkpoint inhibitors, melanoma remains a significant clinical challenge due to its propensity for metastasis, resistance to treatment, and remarkable intratumorally heterogeneity [2, 3]. Recent studies have highlighted the interplay between melanoma progression and metabolic reprogramming, particularly involving short-chain fatty acids (SCFAs), which are microbial-derived metabolites known to influence immune responses and cancer cell behavior [4, 5].

Emerging evidence suggests that SCFAs, such as acetate, propionate, and butyrate, play a dual role in melanoma progression. On one hand, SCFAs can exert anti-tumor effects by modulating immune cell activity and enhancing anti-tumor immunity [6]. On the other hand, melanoma cells may exploit SCFAs as an energy source or signaling molecules to promote survival and proliferation [7]. For instance, butyrate has been shown to inhibit histone deacetylases (HDACs), leading to altered gene expression and potential suppression of tumor growth [8]. However, the precise mechanisms by which SCFAs influence melanoma progression and their interactions with the tumor microenvironment (TME) remain poorly understood.

The heterogeneity of melanoma cells is a hallmark of the disease and a major contributor to its aggressiveness and therapeutic resistance. Melanoma tumors are composed of distinct subpopulations of cancer cells with diverse genetic, epigenetic, and phenotypic profiles [9, 10]. This heterogeneity arises from both intrinsic factors, such as genomic instability and clonal evolution, and extrinsic factors, including interactions with the TME [11]. Single-cell RNA sequencing studies have revealed that melanoma cells can exhibit varying degrees of differentiation, proliferative capacity, and metastatic potential within the same tumor [12, 13]. For example, some subpopulations may express high levels of resistance markers, such as ABC transporters, while others may adopt a stem-like phenotype, contributing to tumor recurrence and metastasis [14]. This cellular diversity not only drives tumor progression but also poses a significant challenge for targeted therapies, as treatment-resistant subclones can survive and repopulate the tumor [15].

The dynamic interplay between melanoma cells and the TME further exacerbates tumor heterogeneity and therapeutic resistance. Among the immune cells within the TME, tumor-associated macrophages (TAMs) play a pivotal role in shaping the tumor landscape. TAMs are known to adopt a pro-tumorigenic (M2-like) phenotype, secreting cytokines and growth factors that enhance tumor cell survival, angiogenesis, and immune evasion [16]. Melanoma cells, in turn, can reprogram TAMs through the secretion of exosomes, cytokines, and metabolites, creating a feedback loop that sustains tumor growth and immune suppression [17, 18]. For instance, melanoma-derived exosomes have been shown to induce macrophage polarization toward an M2-like phenotype, which in turn secretes interleukin-10 (IL-10) and transforming growth factor-beta (TGF-β) to suppress anti-tumor immunity [19]. Additionally, metabolic competition between melanoma cells and macrophages for nutrients such as glucose and SCFAs may further influence tumor progression and immune responses [20].

Despite significant advances in melanoma treatment, including the development of BRAF/MEK inhibitors and immune checkpoint blockers, therapeutic resistance remains a major obstacle. Targeted therapies, such as vemurafenib and dabrafenib, have shown remarkable efficacy in patients with BRAF-mutant melanoma; however, resistance often develops due to the activation of alternative signaling pathways or the emergence of resistant subclones [21]. Similarly, immune checkpoint inhibitors, such as anti-PD-1 and anti-CTLA-4 antibodies, have revolutionized melanoma treatment, but a substantial proportion of patients fail to respond or develop acquired resistance [22, 23]. These limitations underscore the need for a deeper understanding of the molecular and cellular mechanisms driving melanoma progression, including the roles of SCFAs, tumor heterogeneity, and immune cell interactions.

## Methods

### Data acquisition and preprocessing

RNA sequencing (RNA-seq) data from 468 melanoma patients were downloaded from The Cancer Genome Atlas (TCGA) database (https://portal.gdc.cancer.gov/). Raw FASTQ files were quality-checked using FastQC (v0.11.9) and trimmed for adapter sequences and low-quality bases using Trimmomatic (v0.39) with the following parameters: LEADING:3, TRAILING:3, SLIDINGWINDOW:4:15, and MINLEN:36. Cleaned reads were aligned to the human reference genome (GRCh38) using STAR (v2.7.10a) with default parameters. Gene expression quantification was performed using featureCounts (v2.0.1) with the GENCODE v35 annotation file. Batch effects were corrected using the ComBat algorithm in the R package sva (v3.42.0) with default settings. Single-cell RNA sequencing (scRNA-seq) data from the GSE215120 dataset were downloaded from the Gene Expression Omnibus (GEO) database. Raw data were processed using the Seurat R package (v4.3.0). Cells with fewer than 200 detected genes or more than 20% mitochondrial reads were filtered out to remove low-quality cells and potential debris. Data normalization was performed using the LogNormalize method, and scaling was applied to regress out unwanted sources of variation, such as mitochondrial content and cell cycle effects, using the ScaleData function.

### Identification of melanoma subgroups

Non-negative matrix factorization (NMF) clustering was performed using the R package NMF (v0.25.0) to stratify melanoma patients into subgroups based on gene expression profiles. The optimal number of clusters (k = 6) was determined by evaluating the cophenetic coefficient across a range of k values (k = 2 to 10). Short-chain fatty acid (SCFA)-related gene sets (GO:0019745, GO:0019746, and GO:0006085) were downloaded from the Molecular Signatures Database (MSigDB) and used to further refine the clustering. Differential gene expression analysis between subgroups was conducted using the DESeq2 R package (v1.38.0). Genes with a fold change > 2 and an adjusted p-value < 0.05 (Benjamini-Hochberg correction) were considered significantly differentially expressed.

### Functional enrichment analysis

Gene Set Enrichment Analysis (GSEA) was performed using the clusterProfiler R package (v4.6.0) to identify enriched pathways in high-risk melanoma subgroups. The GSEA algorithm was run with 1,000 permutations, and pathways with a false discovery rate (FDR) < 0.05 were considered significantly enriched. KEGG pathway and Gene Ontology (GO) enrichment analyses were conducted using the enrichKEGG and enrichGO functions, respectively, with a significance threshold of FDR < 0.05. Visualization of enriched pathways was performed using the dotplot and cnetplot functions.

### Single-cell RNA sequencing analysis

scRNA-seq data were integrated using the Harmony algorithm (v0.1.0) to correct for batch effects. Principal component analysis (PCA) was performed on the top 2,000 highly variable genes, and the first 30 principal components (PCs) were used for downstream analysis. Cell clusters were identified using the Louvain algorithm with a resolution of 0.5 and annotated based on marker gene expression: cancer-associated fibroblasts (CAFs; PDGFRB, ACTA2), melanocytes/cancer cells (MLANA, TYR), T/NK cells (CD3D, NKG7), dendritic/proliferating cells (CD83, MKI67), endothelial cells (PECAM1, VWF), and macrophages (CD68, CD163). Cell-cell communication analysis was performed using the CellChat R package (v1.6.0). Ligand-receptor pairs were inferred using the default database, and significant interactions were identified with a p-value < 0.05.

### Metabolomics analysis

Non-targeted metabolomics profiling was conducted using liquid chromatography-mass spectrometry (LC-MS) on TEAD3 + melanoma cells treated with GAS6 or PBS. Cells were harvested and metabolites extracted using 80% methanol. LC-MS analysis was performed on a Q Exactive HF-X mass spectrometer (Thermo Fisher Scientific) coupled with a Vanquish UHPLC system. Data were acquired in both positive and negative ionization modes. Metabolites were identified using the MetaboAnalyst 5.0 platform with the HMDB and KEGG databases. Pathway enrichment analysis was performed using the KEGG pathway database, and significantly enriched pathways were defined as those with a p-value < 0.05. Methylmalonic acid levels were quantified using targeted LC-MS with a stable isotope-labeled internal standard (^13^C3-methylmalonic acid). Data were normalized to total protein content measured using the BCA assay (Pierce).

### In vitro functional assays

TEAD3 knockdown in SK-MEL-5. A-375 and MM9H-1 melanoma cells was achieved using three independent siRNAs (Thermo Fisher Scientific, siRNA IDs: s22334, s22335, s22336). Cells were transfected with 50 nM siRNA using Lipofectamine RNAiMAX (Invitrogen) according to the manufacturer’s protocol. Knockdown efficiency was validated by quantitative PCR (qPCR) using SYBR Green Master Mix (Applied Biosystems) and Western blotting with anti-TEAD3 antibody (Abcam, ab133266).

Cell proliferation was assessed using the Cell Counting Kit-8 (CCK-8; Dojindo Laboratories). Briefly, 2,000 cells per well were seeded in 96-well plates, and absorbance at 450 nm was measured at 0, 24, 48, and 72 h.

For Transwell migration and invasion assays, cells were seeded in chambers (8-µm pores; Corning) coated with Matrigel (invasion only; BD Biosciences). After 24 h, membranes were fixed in 4% PFA, stained with 0.1% crystal violet, and imaged at 10 random fields/membrane using a Nikon Eclipse Ti microscope (10× objective). Cell quantification was performed via automated threshold-based analysis (NIS-Elements AR v5.21; object criteria: size > 50 μm², circularity 0.2–1.0), with data expressed as mean cells/field ± SEM.

For scratch assays, confluent monolayers were scratched with a 200-µl pipette tip, imaged at 0 h and 24 h (EVOS M7000, 4× objective), and wound closure (%) was calculated using ImageJ v1.53 as: [1 − (Area_12_ₕ/Area₀ₕ)] × 100%.

Seahorse XF Analyzer (Agilent Technologies) was used to measure oxidative phosphorylation (OCR) and glycolysis (ECAR) rates in GAS6 + and GAS6- macrophages. Cells were seeded in XF24 cell culture plates and incubated in XF assay medium (Agilent) for 1 h prior to analysis.

ATP levels were quantified using the ATP Assay Kit (Abcam, ab83355) according to the manufacturer’s instructions.

### TEAD3-KO B16F10luc cell generation

TEAD3-knockout (KO) B16F10luc melanoma cells were generated using CRISPR/Cas9 genome editing. Two high-efficiency single-guide RNAs (sgRNAs) targeting TEAD3 exons were designed via the CRISPR Design Tool (http://crispr.mit.edu/) with the following sequences:

sgRNA1: 5′-GACCTGCGCAAGATCCTGCT-3′ (Exon 2).

sgRNA2: 5′-GTCATGGCTCCGTACCCGAG-3′ (Exon 4).

Oligonucleotides were annealed and cloned into BbsI-linearized pSpCas9(BB)-2 A-Puro (Addgene #62988) using T4 DNA ligase. B16F10luc cells (70% confluent in 6-well plates) were co-transfected with 2 µg sgRNA plasmids using Lipofectamine 3000 (Thermo Fisher). After 48 h, puromycin selection (2 µg/mL) was applied for 72 h. Surviving cells were single-cell sorted by FACS into 96-well plates. Knockout efficiency was confirmed by qPCR and Western blotting.

### Myeloid-specific GAS6 knockout mice model

Myeloid-specific GAS6 knockout mice were generated by crossing homozygous GAS6^fl/fl^ mice (Jackosn lab #026554) with heterozygous LysM-Cre mice (Jackson lab #004781) on a C57BL/6J background. GAS6^fl/fl^ females were bred with LysM-Cre^+/−^males to generate GAS6^fl/fl^;LysM-Cre^+/−^ experimental mice and GAS6^fl/fl^;Cre^−/−^ littermate controls. Genotyping was performed by PCR of tail DNA.

### In vivo experiments

For tumor growth assays, 1 × 10^6 TEAD3-KO or wild-type (WT) B16F10luc cells were subcutaneously injected into the flanks of 6- to 8-week-old C57BL/6 mice (*n* = 6 per group). Similarly, for the YUMMER1.7 syngeneic model, 1.5 × 10^6 TEAD3-KO or WT YUMMER1.7 cells were injected subcutaneously into C57BL/6 mice (*n* = 6 per group). Bioluminescent imaging was performed using the IVIS Spectrum system (PerkinElmer) after intraperitoneal injection of D-luciferin (150 mg/kg). For metastasis assays, 5 × 10^5 B16F10luc or YUMMER1.7 cells were injected intravenously via the tail vein, and lung metastases were quantified at day 21. Anti-PD-1 therapy (10 mg/kg, Bio X Cell, BE0146) was administered intraperitoneally twice weekly for 3 weeks.

### Assessment of treatment-related adverse effects

To comprehensively evaluate potential toxicities associated with therapeutic targeting of the GAS6-TYRO3 axis and its combination with anti-PD-1 therapy, we implemented a multi-parameter toxicity assessment protocol. Body weight was monitored every 3 days using a calibrated scale (Sartorius CPA225D, ± 0.01 g sensitivity). Serum biomarkers of organ dysfunction were analyzed at endpoint: liver function (ALT/AST; Sigma-Aldrich MAK052), renal function (BUN/creatinine; Abcam ab83362), and pancreatic injury (amylase/lipase; Invitrogen EIAMYL/EIAMSG) were quantified via commercial ELISA kits. Hematological profiles (complete blood counts with differentials) were assessed using an automated hematology analyzer (Sysmex XN-1000).

### Immunohistochemistry (IHC) and flow cytometry

Formalin-fixed, paraffin-embedded tumor tissues were sectioned at 4 μm thickness and stained for TEAD3 (Abcam, ab133266), N-cadherin (Cell Signaling Technology, #13116), and E-cadherin (Cell Signaling Technology, #3195) using standard IHC protocols. Antigen retrieval was performed in citrate buffer (pH 6.0) at 95 °C for 20 min. Sections were incubated with primary antibodies overnight at 4 °C, followed by HRP-conjugated secondary antibodies (Dako) and DAB substrate (Vector Laboratories). For flow cytometry, single-cell suspensions from tumor tissues were prepared by mechanical dissociation and enzymatic digestion with collagenase IV (1 mg/mL, Sigma) for 30 min at 37 °C. Cells were stained with antibodies against CD3 (BioLegend, 100204), CD8 (BioLegend, 100708), CD163 (BioLegend, 333602), and TIGIT (BioLegend, 151702) for 30 min at 4 °C. Data were acquired using a BD LSRFortessa flow cytometer and analyzed using FlowJo software (v10.8.1).

### Statistical analysis

All statistical analyses were performed using R (v4.2.2) or GraphPad Prism (v9.0). Data are presented as mean ± standard deviation (SD). Comparisons between two groups were performed using Student’s t-test, and multiple group comparisons were analyzed using one-way ANOVA followed by Tukey’s post hoc test. Survival analysis was conducted using the Kaplan-Meier method, and differences were assessed using the log-rank test. A p-value < 0.05 was considered statistically significant.

## Results

### Identification of melanoma subgroups based on Short-Chain fatty acid metabolism and their association with clinical outcomes

**Fig. 1:**
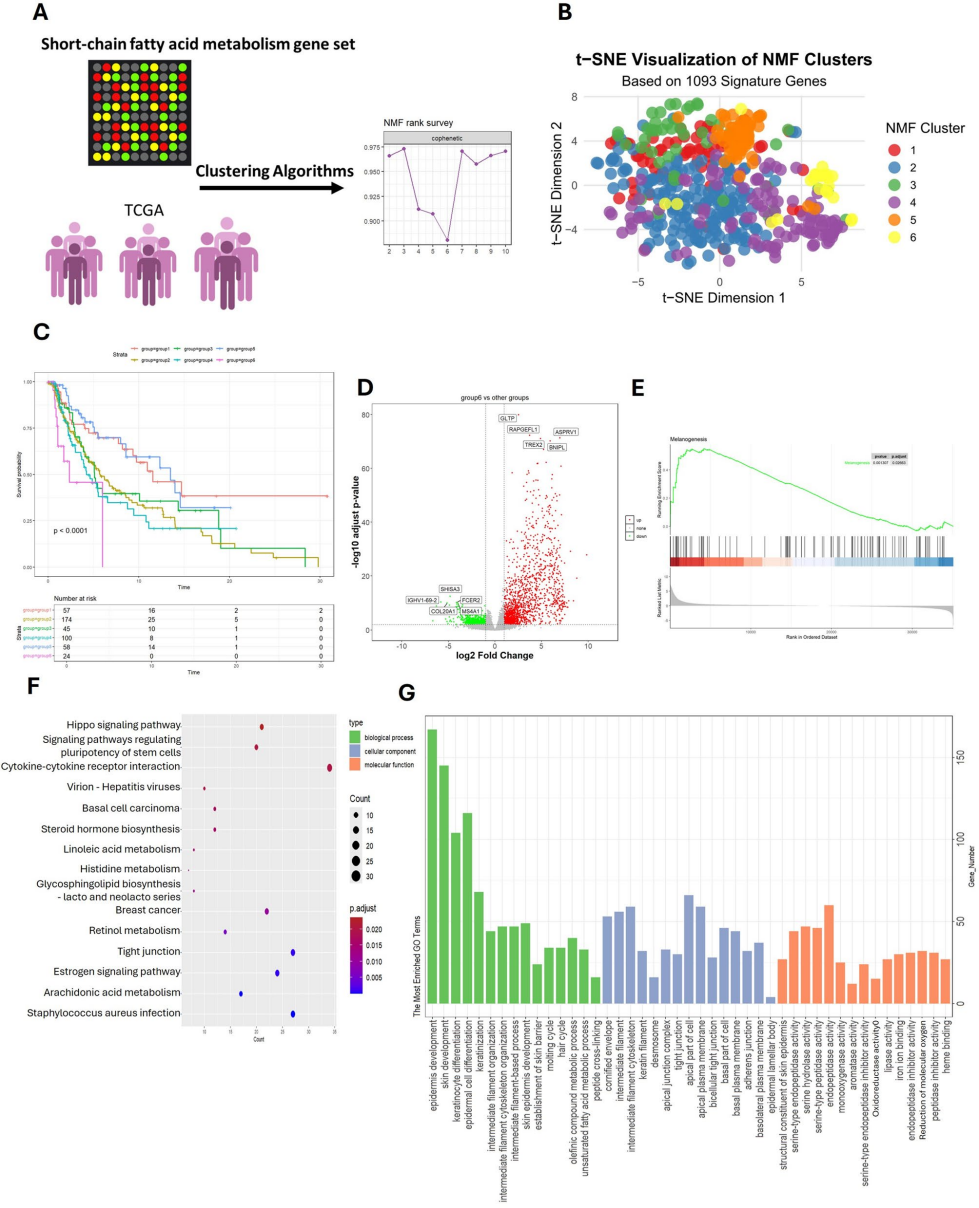
Identification of SCFA-Driven Melanoma Subgroups with Distinct Clinical Outcomes (**A**) NMF clustering of 468 TCGA melanoma samples using SCFA-related gene sets (GO:0019745, GO:0019746, GO:0006085) identified six molecular subgroups. The optimal cluster number (k = 6) was determined by cophenetic coefficient stability. (**B**) TSNE map of SCFA-related gene expression across subgroups, with Group 6 showing unique gathering. (**C**) Kaplan-Meier survival curves demonstrating significantly worse overall survival in Group 6. (**D**) Volcano plot of differential gene expression (Group 6 vs. others) highlighting upregulated GLTP and RAPGEFL1. (**E-G**) Functional enrichment analysis of Group 6: (**E**) GSEA melanogenesis pathway enrichment (FDR < 0.25), (**F**) KEGG pathway analysis and (**G**) GO terms

To investigate the heterogeneity of melanoma and its association with lipid metabolism, we analyzed RNA sequencing data from 468 melanoma patients obtained from The Cancer Genome Atlas (TCGA) database. Using the non-negative matrix factorization (NMF) algorithm implemented in the R package NMF, we performed clustering analysis and selected six clusters based on the cophenetic coefficient (Fig. 1A). To further explore the role of lipid metabolism, we utilized three short-chain fatty acid (SCFA)-related gene sets (GO:0019745 - Short-chain fatty acid metabolic process, GO:0019746 - Propanoate metabolism, and GO:0006085 - Acetate metabolic process) comprising 1,093 genes and stratified the patients into six distinct groups (Fig. 1B).

Kaplan-Meier survival analysis of the six RNAseq-based clusters revealed that patients in Group 6 exhibited significantly worse overall survival compared to the other groups (*P* < 0.0001, Fig. 1C). To identify potential drivers of this poor prognosis, we performed differential gene expression analysis between Group 6 and the other groups. Notably, genes such as GLTP and RAPGEFL1 were significantly upregulated in Group 6 (Fig. 1D).

To gain further insights into the biological processes associated with Group 6, we conducted functional enrichment analysis on the 1,568 upregulated genes identified in this group. Gene Set Enrichment Analysis (GSEA) revealed significant enrichment of the Melanogenesis pathway in Group 6 (Fig. 1E). KEGG pathway analysis highlighted several key pathways, including Hippo signaling pathway, Cytokine-cytokine receptor interaction, Linoleic acid metabolism, and Retinol metabolism (Fig. 1F). Additionally, Gene Ontology (GO) enrichment analysis demonstrated significant associations with skin-related processes such as epidermis development, skin development, and keratinocyte differentiation, as well as lipid metabolism-related terms such as lipase activity (Fig. 1G).

These findings suggest that Group 6 represents a distinct molecular subtype of melanoma characterized by dysregulated lipid metabolism, activation of melanogenesis-related pathways, and poor clinical outcomes. The upregulation of genes such as GLTP and RAPGEFL1, along with the enrichment of pathways involved in skin development and lipid metabolism, may provide potential therapeutic targets for this high-risk subgroup.

### Identification of TEAD3 as a key risk gene driving melanoma progression and poor prognosis

**Fig. 2:**
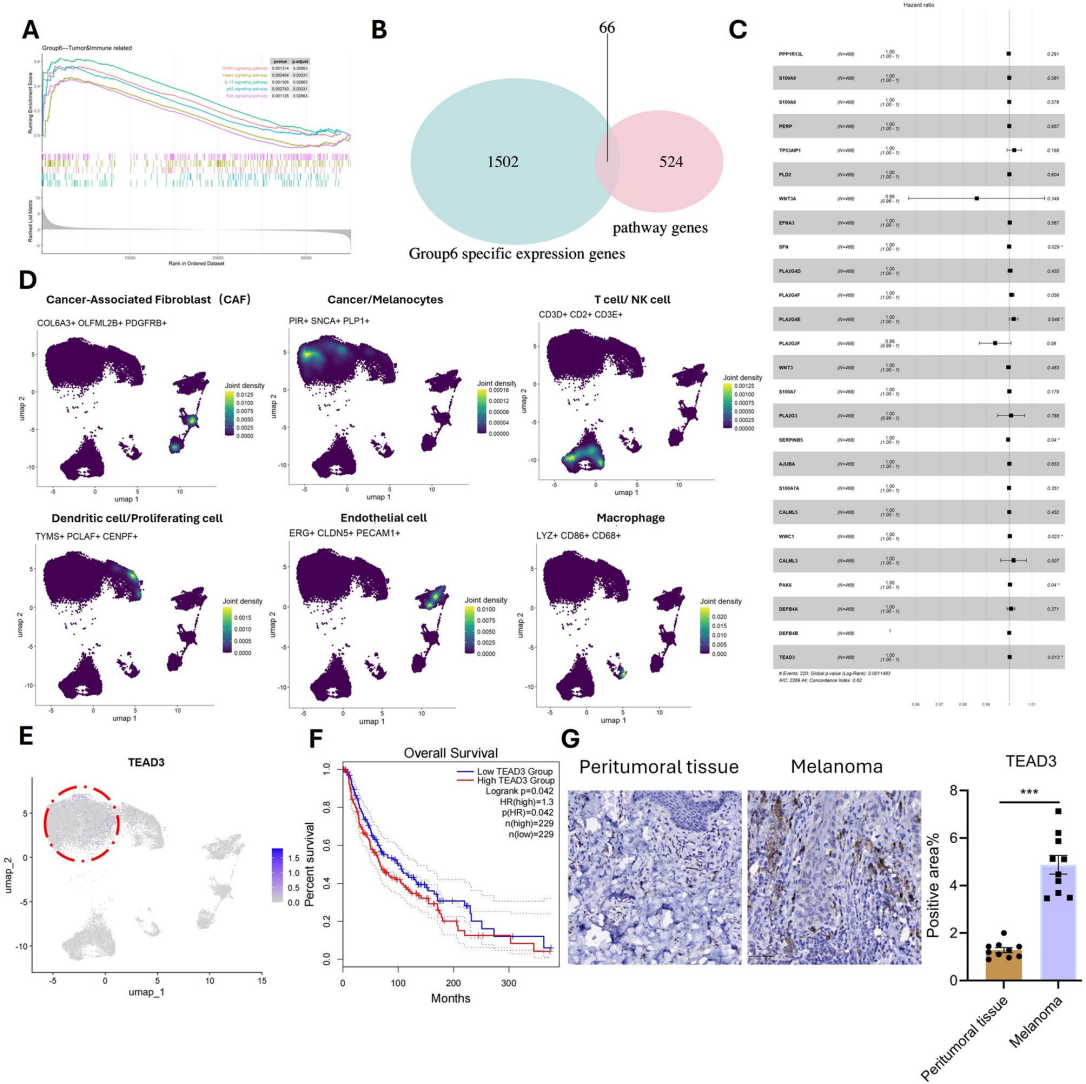
TEAD3 Emerges as a Key Driver of Melanoma Progression. (**A**) GSEA of Group 6 showing enriched cancer-related pathways. (**B**) Venn diagram intersecting pathway genes with Group 6 upregulated genes, yielding 66 candidates. (**C**) Forest plot of Cox regression risk scores identifying TEAD3, SFN, and PAK6 as top prognostic genes. (**D**) Single-cell RNA-seq analysis (GSE215120): cell type annotation. (**E**) Single-cell expression of risk genes (TEAD3 tumor-specific). (**F**) High TEAD3 expression correlates with poor survival in TCGA (log-rank *P* = 0.042). (**G**) IHC staining of TEAD3 in peritumoral tissue and tumor

To further elucidate the molecular mechanisms driving the poor prognosis of Group 6 melanoma patients, we performed Gene Set Enrichment Analysis (GSEA) and identified several immune- and cancer-related pathways significantly enriched in this subgroup, including GnRH signaling pathway, Hippo signaling pathway, IL-17 signaling pathway, p53 signaling pathway, and Ras signaling pathway (Fig. 2A). We intersected the genes from these pathways with the 1,568 upregulated genes in Group 6, resulting in 66 overlapping genes (Fig. 2B). These genes were subsequently subjected to a COX risk model analysis, which identified 26 risk genes significantly associated with melanoma progression: PPP1R13L, S100A9, S100A8, PERP, TP53AIP1, PLD2, WNT3A, EFNA3, SFN, PLA2G4D, PLA2G4F, PLA2G4E, PLA2G2F, WNT3, S100A7A, CALML5, WWC1, CALML3, PAK6, DEFB4A, DEFB4B, and TEAD3.

Risk forest plot analysis revealed that SFN, PLA2G4E, SERPINB5, WWC1, PAK*6*, and *TEAD3* were the most influential genes in promoting melanoma malignancy (Fig. 2C). To further investigate the functional roles of these risk genes, we analyzed the data from acral melanoma patients (GSE215120 dataset). Cells were clustered using UMAP visualization and annotated into distinct populations based on established marker gene expression profiles. This analysis identified cancer-associated fibroblasts (CAFs), melanocytes/cancer cells, T cells/NK cells, dendritic cells/proliferating cells, endothelial cells, and macrophages (Fig. 2D). Single-cell localization analysis of the risk genes demonstrated that only *ADH7*, *SFN*, *WWC1*, *PAK6*, and *TEAD3* were detectable in the single-cell data, with *TEAD3* being predominantly expressed in tumor cells (Fig. 2E).

Consistent with these findings, survival analysis using TCGA melanoma data revealed that high *TEAD3* expression was significantly associated with poorer overall survival (*P* = 0.042, Fig. 2F). To validate these results at the protein level, we performed immunohistochemical staining for TEAD3 in human melanoma and adjacent normal tissues. The results confirmed that TEAD3 was highly expressed in tumor tissues compared to normal tissues (Fig. 2G), further supporting its role as a potential driver of melanoma progression.

### Functional validation of TEAD3 as a critical driver of acral melanoma progression in vitro and in vivo

**Fig. 3:**
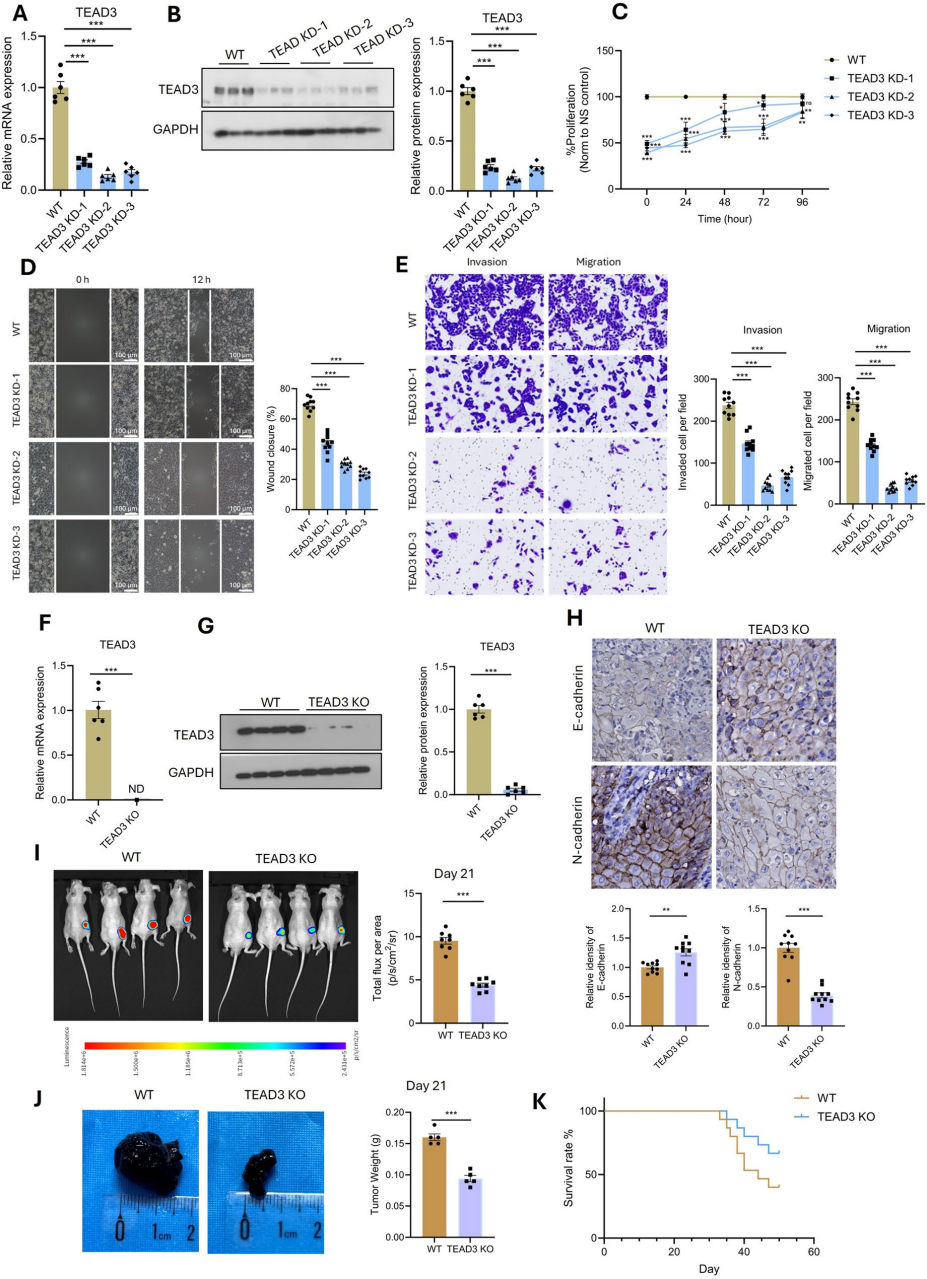
TEAD3 Knockout Suppresses Melanoma Growth and EMT. (**A-B**) siRNA-mediated TEAD3 knockdown in SK-MEL-5 cells: (**A**) qPCR, (**B**) Western blot. (**C-E**) Functional assays post-knockdown: (**C**) Proliferation (CellTiter-Glo), (D) Migration (Transwell), (**E**) Invasion (Matrigel). (**F-G**) CRISPR/Cas9 TEAD3 knockout in B16F10luc cells: (**F**) qPCR, (**G**) Western blot. (**H**) IHC of EMT markers (N-cadherin↓, E-cadherin↑) in TEAD3-KO tumors. (**I-J**) In vivo tumor growth: (I) Bioluminescence imaging, (**J**) Tumor volume. (**K**) Survival curves of mice bearing WT vs. TEAD3-KO tumors

To validate the functional role of TEAD3 in melanoma progression, we performed a series of in vitro and in vivo experiments using multiple melanoma cell lines and syngeneic mouse models. Initially, we designed three independent siRNAs targeting TEAD3 and confirmed their knockdown efficiency in the SK-MEL-5 human melanoma cell line. Quantitative PCR and Western blot analyses demonstrated that all three siRNAs significantly reduced TEAD3 mRNA (Fig. 3A) and protein levels (Fig. 3B). Functional assays revealed that TEAD3 knockdown markedly inhibited melanoma cell proliferation (Fig. 3C), migration as measured by horizontal cell movement (Fig. 3D), and invasion through extracellular matrix barriers (Fig. 3E), suggesting that TEAD3 plays a critical role in promoting melanoma cell growth and metastatic potential.

To further generalize these findings, we extended the TEAD3 knockdown experiments to two additional melanoma cell lines, A-375 and MM9H-1. Consistent with the results in SK-MEL-5 cells, TEAD3 knockdown in both A-375 and MM9H-1 cells led to significant suppression of proliferation, migration, and invasion (Supplementary Figure S1A–F), reinforcing the crucial role of TEAD3 across different genetic backgrounds of melanoma.

To investigate the impact of TEAD3 loss in vivo, we first generated a TEAD3-knockout (KO) B16F10luc cell line using CRISPR/Cas9 technology. The knockout efficiency was confirmed by qPCR and WB, which showed a complete absence of TEAD3 mRNA and protein expression in the KO cells (Figs. 3F–G). Subcutaneous injection of TEAD3-KO and wild-type (WT) cells into mice revealed that TEAD3 deficiency significantly reduced tumor growth, as evidenced by lighter tumor weight (Fig. 3J) and decreased bioluminescent signal intensity in live animal imaging (Fig. 3I). Immunohistochemical (IHC) analysis of tumor tissues harvested on day 21 demonstrated that TEAD3-KO tumors exhibited reduced epithelial-mesenchymal transition (EMT), characterized by downregulation of N-cadherin and upregulation of E-cadherin (Fig. 3H).

To further evaluate the role of TEAD3 in a more clinically relevant and immunocompetent microenvironment, we utilized the YUMMER1.7 syngeneic mouse model, which closely mimics key characteristics of human immunotherapy-resistant melanoma. Similarly, TEAD3-KO YUMMER1.7 cells were generated via CRISPR/Cas9, and knockout was verified at both mRNA and protein levels (Supplementary Figure S2A–B). Upon implantation into syngeneic mice, TEAD3 deficiency led to a pronounced reduction in tumor growth (Supplementary Figure S2C–D) and attenuated EMT markers in IHC analysis (Supplementary Figure S2E), corroborating the results obtained with the B16F10luc model.

Finally, survival analysis showed that mice bearing TEAD3-KO tumors in both the B16F10luc and YUMMER1.7 models had significantly longer overall survival compared to those with WT tumors (Fig. 3K and Supplementary Figure S2F). Collectively, these results demonstrate that TEAD3 is a critical regulator of melanoma progression across multiple cellular contexts and in vivo models, influencing tumor growth, invasion, and metastasis. Its role in promoting EMT and its association with poor survival outcomes highlight TEAD3 as a potential therapeutic target for melanoma treatment.

### TEAD3 expression promotes tumor malignancy via M2 macrophage crosstalk and GAS6-TYRO3 signaling in a spatially defined microenvironment

**Fig. 4:**
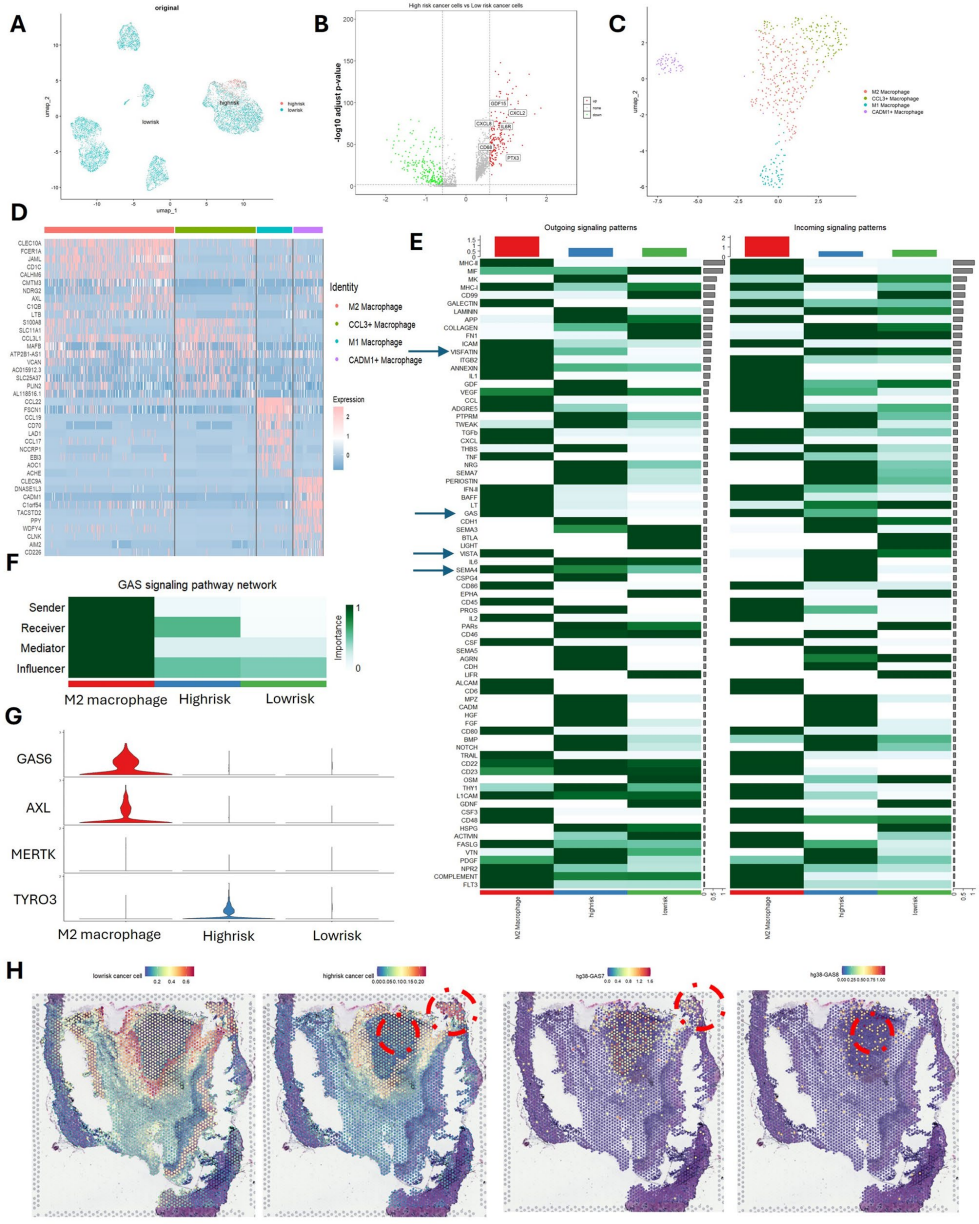
TEAD3 Drives Tumor-M2 Macrophage Crosstalk via GAS6-TYRO3 in a Spatial Niche. (**A**) Single-cell RNA-seq-based stratification of melanoma cells into high-risk (TEAD3-high) and low-risk (TEAD3-low) subgroups (UMAP). (**B**) Volcano plot of differentially expressed genes in high-risk vs. low-risk cells, highlighting M2 macrophage-associated markers (CXCL2, IL6R, CD68). (**C**) UMAP of macrophage subpopulations extracted from single-cell data. (**D**) Macrophage subtype annotation. (**E**) Cell-cell communication analysis (CellChat) showing M2 macrophage-derived signaling pathways enriched in high-risk tumor cells. (**F-G**) Heatmap of ligand-receptor pairs: (**F**) GAS pathway interactions, (**G**) GAS6-TYRO3 as the top candidate. (**H**) Spatial transcriptomics mapping of high-risk tumor cell clusters (left) co-localized with GAS6/GAS7-enriched niches (right)

To further elucidate the reasons behind the high malignancy of tumor cells with elevated expression of TEAD3, we isolated tumor cells from single-cell data and categorized them into high-risk and low-risk cancer cells based on their TEAD3 expression levels (Fig. 4A). Differential analysis between high-risk and low-risk cancer cells revealed that high-risk cancer cells exhibited elevated expression of genes associated with M2 macrophages, including CXCL2, IL6R, CXCL8, GDF15, CD68, and PTX3 (Fig. 4B). This suggests a strong correlation between high-risk cancer cells and M2 macrophages. To validate this hypothesis, we extracted single-cell data of macrophages and classified them into subtypes (Fig. 4C and D), and subsequently focused on M2 macrophages, high-risk cancer cells, and low-risk cancer cells for cell-cell communication analysis. The results demonstrated that signaling pathways such as VISFATIN, VISTA, GAS, and SEMA4 were active in M2 macrophages and exclusively received by high-risk cancer cells (Fig. 4E). Further analysis of ligand-receptor pairs revealed that VTN-(ITGAV + ITGB5), SEMA4D-PLXNB2, GAS6-TYRO3, and FN1-(ITGA3 + ITGB1) were more prevalent in high-risk cancer cells. To identify the most likely signaling pathway involved in this communication, we conducted a detailed analysis of the significant interactions. Through heatmap analysis of the GAS pathway and examination of its ligand-receptor pairs (Figs. 4F), as well as violin plots showing the expression levels of GAS pathway genes (Fig. 4G), the GAS6-TYRO3 ligand-receptor pair emerged as a strong candidate. Since single-cell data lacks spatial distribution information, we integrated single-cell and spatial transcriptomic data to spatially map low-risk and high-risk cancer cells. High-risk cancer cells were found to form clusters with a unique spatial niche. Spatial localization of GAS6 and GAS7 expressions within the GAS pathway revealed concentrated expression of these genes in regions populated by high-risk cancer cells (Fig. 4H). These findings suggest that the GAS6-TYRO3 interaction may play a critical role in the malignant behavior of high-risk cancer cells, potentially mediated through their spatial organization and communication with M2 macrophages.

### Hypermetabolic GAS6 + macrophages enhance GAS6 secretion via oxidative phosphorylation and glycolysis in the tumor microenvironment

**Fig. 5:**
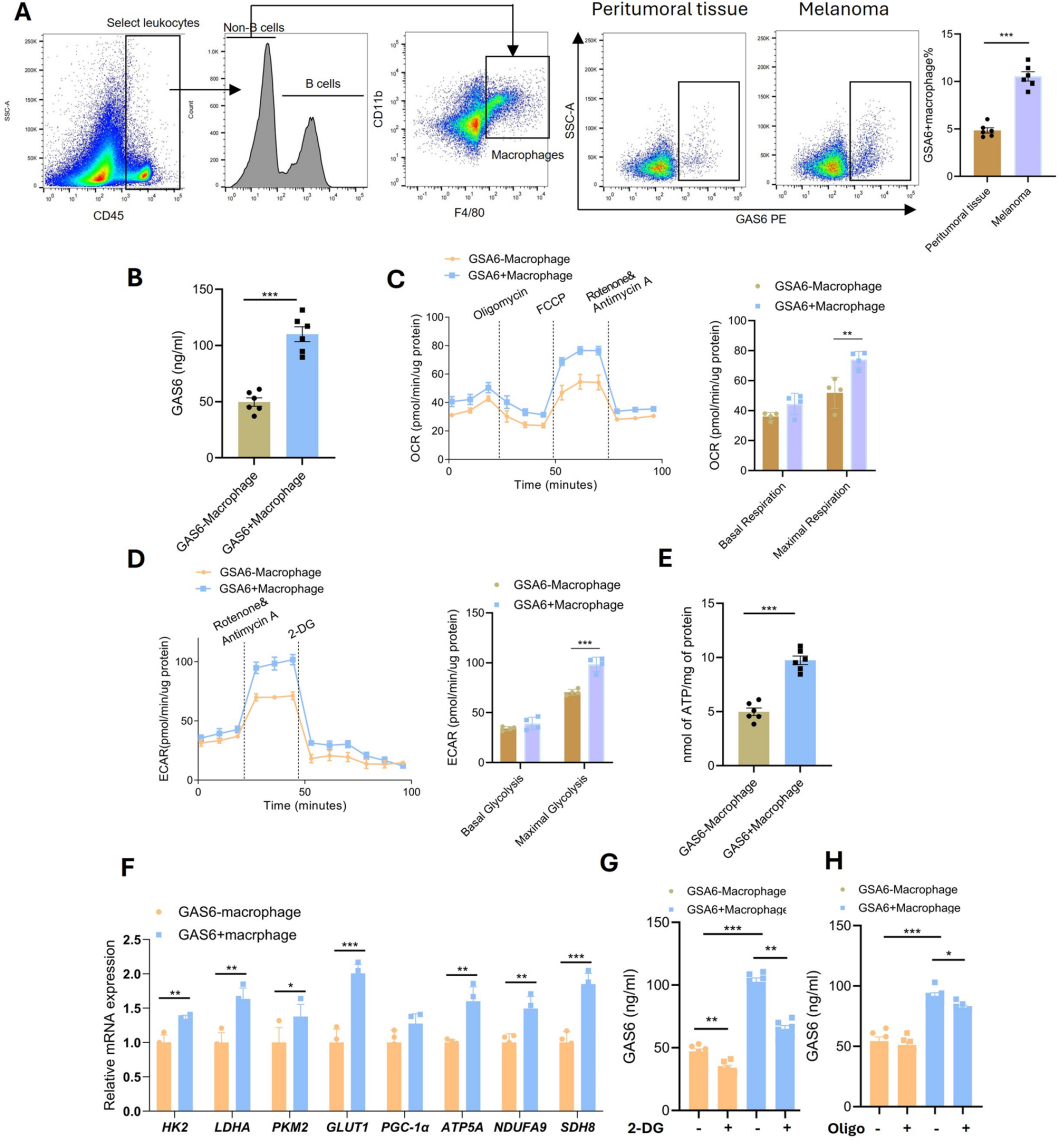
Hypermetabolic GAS6 + Macrophages Drive GAS6 Secretion. (**A**) Flow cytometry showing increased GAS6 + macrophages in tumors vs. normal tissues. (**B**) ELISA confirming elevated GAS6 secretion by GAS6 + macrophages (**P < 0.001). (**C-D**) Seahorse assays: (**C**) Glycolysis (ECAR), (**D**) OXPHOS (OCR) in GAS6 + vs. GAS6- macrophages. (**E**) ATP levels in GAS6 + macrophages. (**F**) qPCR of glycolysis/OXPHOS genes. (**G-H**) Inhibitor treatment (2-DG, Oligomycin) reduces GAS6 secretion

To validate the single-cell RNA sequencing results, we isolated GAS6 + macrophages from B16F10luc-induced syngeneic melanoma tumors and adjacent normal tissues using flow cytometry, revealing a significantly higher proportion of GAS6 + macrophages in tumor tissues (Fig. 5A). Next, we cultured GAS6- and GAS6 + macrophages in vitro and measured GAS6 secretion by ELISA, demonstrating that GAS6 + macrophages secreted significantly higher levels of GAS6 (Fig. 5B). Given that increased ligand secretion often requires enhanced energy metabolism, we assessed the metabolic activity of GAS6 + macrophages using the Seahorse XF Analyzer, which showed significantly elevated oxidative phosphorylation (OXPHOS) and glycolysis rates in GAS6 + macrophages (Fig. 5C, D), accompanied by increased ATP production (Fig. 5E). qPCR analysis further confirmed upregulation of OXPHOS- and glycolysis-related genes in GAS6 + macrophages (Fig. 5F). To establish a functional link between energy metabolism and GAS6 secretion, we treated GAS6 + macrophages with the glycolysis inhibitor 2-DG and the OXPHOS inhibitor Oligomycin, both of which significantly reduced GAS6 secretion (Fig. 5G, H). These findings collectively demonstrate that GAS6 + macrophages exhibit a hypermetabolic phenotype, which drives GAS6 secretion and likely contributes to tumor progression.

### The GAS6-TYRO3 axis mediates macrophage-melanoma crosstalk to drive tumor proliferation, EMT, and metastasis

**Fig. 6:**
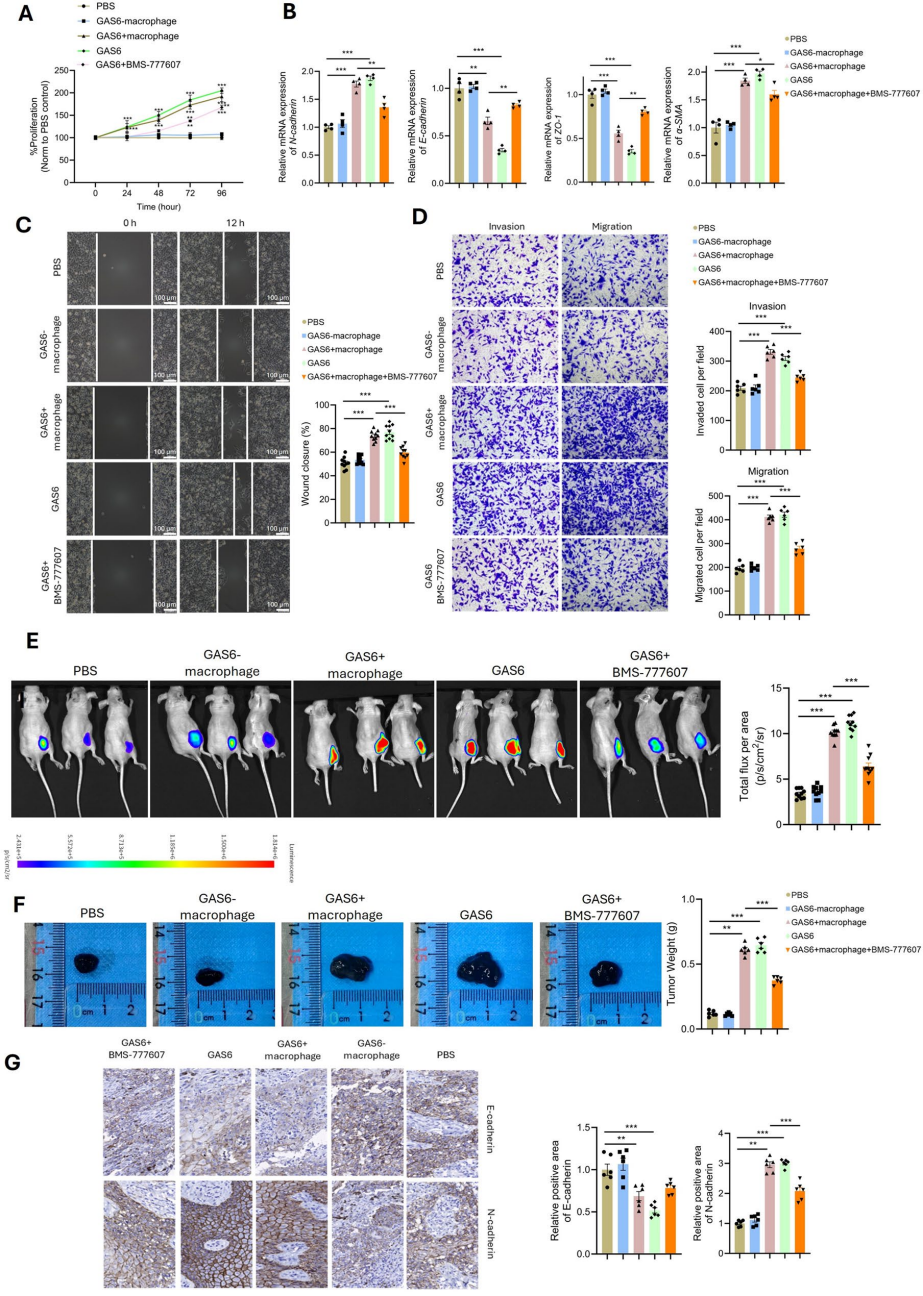
GAS6-TYRO3 Axis Promotes Tumor Proliferation and EMT. (**A**) Co-culture with GAS6 + macrophages enhances melanoma proliferation. (**B**) qPCR of EMT markers (N-cadherin↑, E-cadherin↓) in GAS6-treated cells. (**C-D**) Functional assays: (**C**) Scratch wound healing, (**D**) Invasion (Matrigel). (**E-F**) In vivo tumor growth: (**E**) Fluorescence intensity, (**F**) Tumor weight (**P < 0.001). (**G**) IHC of EMT markers in tumors co-cultured with GAS6 + macrophages

To investigate the role of the GAS6-TYRO3 axis in mediating crosstalk between TEAD3 + melanoma cells and macrophages, we isolated TEAD3 + high-risk tumor cells from melanoma-bearing mice and established five experimental groups: [1] PBS-treated control [2], co-culture with GAS6- macrophages [3], co-culture with GAS6 + macrophages [4], treatment with GAS6 alone, and [5] co-culture with GAS6 + macrophages in the presence of the TYRO3 inhibitor BMS-777,607. After 24 h of co-culture, tumor cell proliferation was significantly enhanced in the GAS6 + macrophage co-culture and GAS6 treatment groups compared to the PBS and GAS6- macrophage co-culture groups, while TYRO3 inhibition suppressed this effect (Fig. 6A). Additionally, GAS6 + macrophage co-culture and GAS6 treatment upregulated EMT-related genes (N-cadherin and alpha-SMA) and downregulated epithelial markers (E-cadherin and ZO-1), which were partially reversed by TYRO3 inhibition (Fig. 6B). Functional assays, including scratch wound healing, invasion, and migration experiments, further demonstrated that GAS6 + macrophage co-culture and GAS6 treatment significantly enhanced tumor cell motility and invasiveness, while BMS-777,607 treatment abolished these effects (Fig. 6C, D). In vivo, intravenous injection of treated melanoma cells revealed that GAS6 + macrophage co-culture and GAS6 treatment significantly increased tumor fluorescence intensity and weight compared to controls, whereas TYRO3 inhibition suppressed tumor growth (Fig. 6E, F). Immunohistochemical (IHC) analysis of tumor tissues confirmed that GAS6 + macrophage co-culture and GAS6 treatment promoted EMT (increased N-cadherin and decreased E-cadherin), which was attenuated by TYRO3 inhibition (Fig. 6G). These findings demonstrate that the GAS6-TYRO3 axis mediates crosstalk between macrophages and TEAD3 + melanoma cells, driving tumor proliferation, EMT, invasion, and metastasis.

### GAS6 drives TEAD3 + melanoma cell aggressiveness by reprogramming propionate metabolism via mmut-mediated methylmalonic acid accumulation

**Fig. 7:**
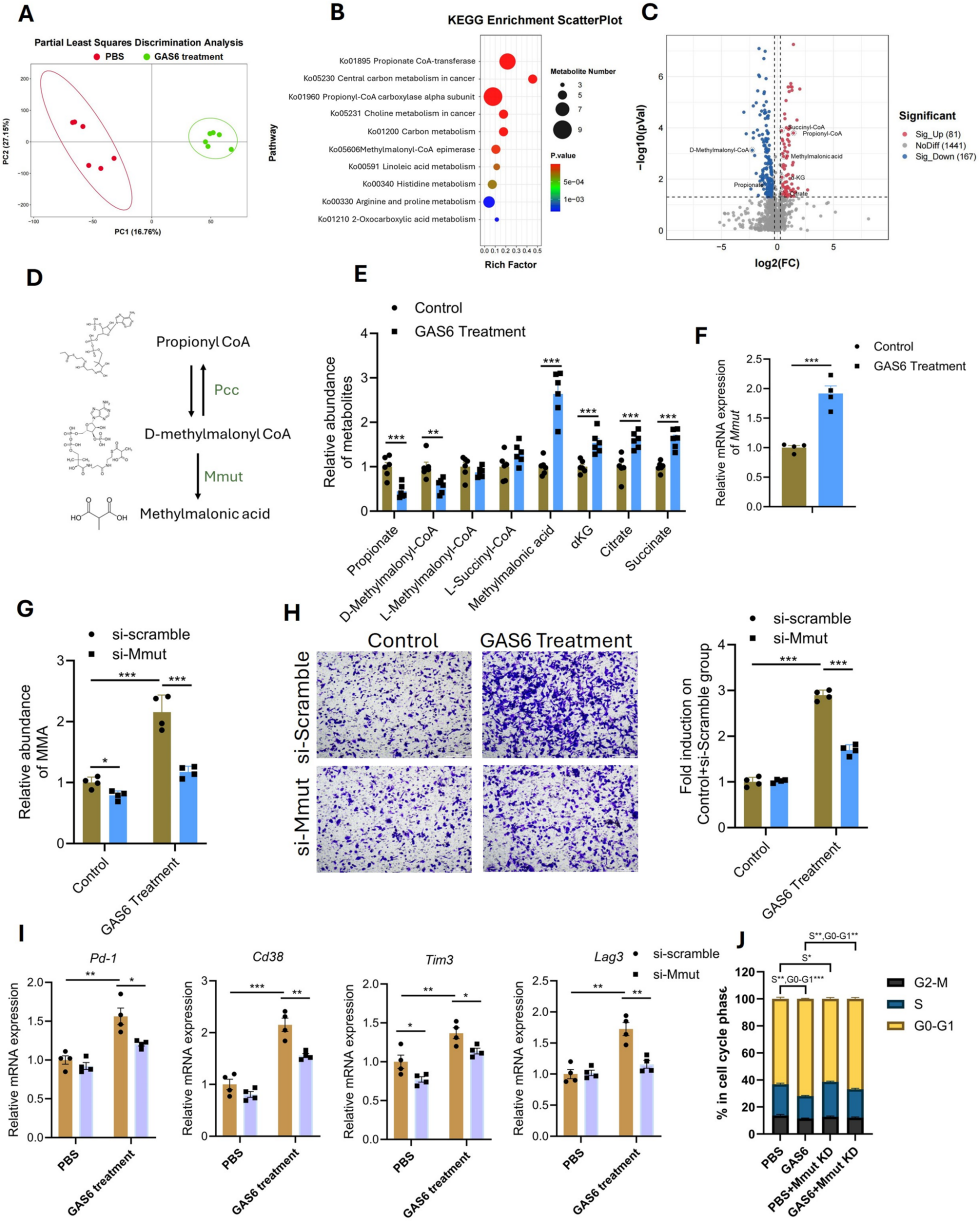
GAS6 Reprograms Propionate Metabolism via Mmut. (**A**) PCA of metabolomics data separates GAS6-treated vs. control TEAD3 + cells. (**B**) KEGG enrichment showing propionate metabolism pathways (FDR < 0.05). (**C**) Volcano plot of propionate intermediates (methylmalonic acid↑, propionate↓). (**D**) The catabolic pathway from propionyl CoA to methylmalonic acid. (**E**) LC/MS validation of methylmalonic acid accumulation. (**F**) GAS6 treatment increases the mRNA level of Mmut. (**G-H**) Mmut knockdown reduces methylmalonic acid and reverses GAS6-induced invasion. (**I**) Conditioned medium (CM) was collected from TEAD3 + tumor cells subjected to four treatments. Primary murine CD8⁺ T cells were cultured with the respective CM, and expression levels of exhaustion markers (PD-1, CD38, TIM-3, LAG-3) were quantified by QPCR. (**J**) Cell cycle distribution of CD8⁺ T cells after CM exposure

To investigate how GAS6 secretion interacts with TEAD3 + melanoma cells, we isolated TEAD3 + tumor cells from a B16F10luc melanoma mouse model and treated them with either GAS6 or PBS (control) during vitro culture, followed by non-targeted metabolomics sequencing. Principal Component Analysis (PCA) revealed a clear separation between the GAS6-treated and control groups, indicating significant differences in their metabolic profiles (Fig. 7A). KEGG pathway enrichment analysis identified three propionate metabolism-related pathways (propionate-CoA transferase, propionyl-CoA carboxylase alpha subunit, and methylmalonyl-CoA epimerase) among the top 10 enriched pathways (Fig. 7B). Volcano plot analysis further highlighted significant changes in key intermediates of propionate metabolism, with increased levels of succinyl-CoA, propionyl-CoA, methylmalonic acid, alpha-ketoglutarate (α-KG), and citrate, and decreased levels of D-methylmalonyl-CoA and propionate in GAS6-treated cells (Fig. 7C). Notably, methylmalonic acid (MMA), a metabolite recently linked to cancer cell proliferation and aggressiveness [34, 35], was significantly accumulated in GAS6-treated cells, as validated by LC/MS (Fig. 7D and E). This accumulation was driven by GAS6-induced upregulation of D-methylmalonyl-CoA hydrolase (Mmut), as confirmed by qPCR (Fig. 7F). Knockdown of Mmut in TEAD3 + melanoma cells significantly reduced methylmalonic acid levels in both PBS- and GAS6-treated groups (Fig. 7G). Functional assays demonstrated that Mmut knockdown reversed the GAS6-induced enhancement of migration in TEAD3 + melanoma cells (Fig. 7H), indicating that GAS6 promotes melanoma cell aggressiveness by dysregulating propionate metabolism through Mmut upregulation and methylmalonic acid accumulation.

To further explore whether GAS6-induced MMA accumulation contributes to immunosuppression—a key rationale for targeting this pathway—we collected conditioned medium (CM) from four treatment groups of TEAD3 + tumor cells and co-cultured it with primary murine CD8⁺ T cells. We found that CM from GAS6-treated cells significantly upregulated exhaustion markers (PD-1, CD38, TIM-3, LAG-3) and induced cell cycle arrest in the G0-G1 phase in CD8⁺ T cells (Fig. 7I and J). Importantly, these effects were markedly reversed when CM from Mmut-knockdown cells—which reduces MMA accumulation—was used (Fig. 7I and J). These data demonstrate that GAS6-driven MMA accumulation directly impairs CD8⁺ T cell function by promoting exhaustion and cell cycle arrest, providing a mechanistic basis for the enhanced efficacy of anti-PD-1 therapy in combination with Mmut or GAS6 targeting.

Collectively, these findings reveal a critical metabolic-immune crosstalk whereby GAS6 reprograms propionate metabolism in TEAD3 + melanoma cells via Mmut-mediated MMA accumulation, which not only enhances cell aggressiveness but also fosters an immunosuppressive microenvironment. This underscores Mmut and methylmalonic acid as promising therapeutic targets to disrupt both tumor-intrinsic and immune-suppressive mechanisms in melanoma.

### Myeloid-GAS6 knockout enhances Anti-PD-1 therapy by boosting antitumor immunity and improving melanoma treatment outcomes

**Fig. 8:**
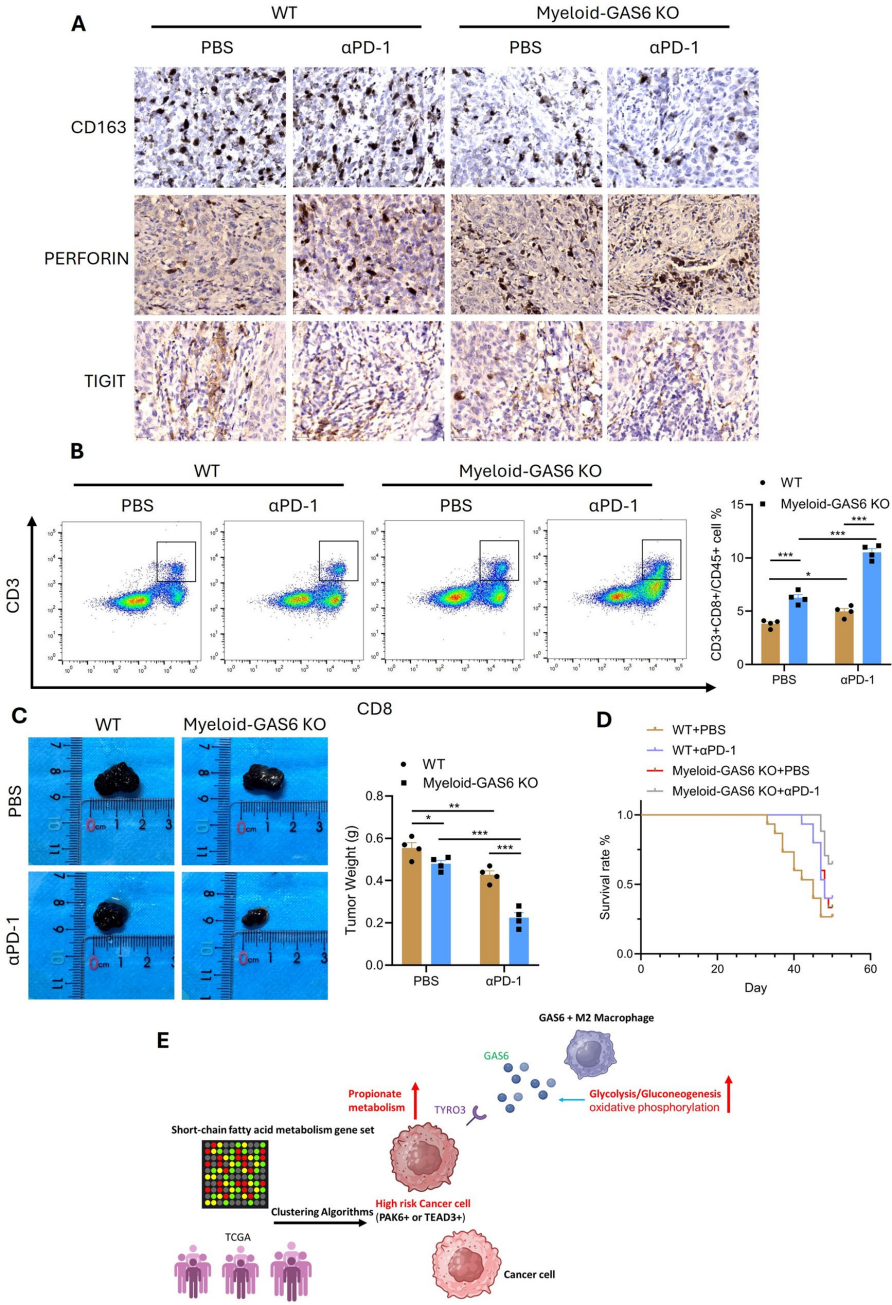
Myeloid-GAS6 Knockout Synergizes with Anti-PD-1 Therapy. (**A**) IHC of immunosuppressive (CD163, TIGIT↓) and cytotoxic (perforin↑) markers. (B**)** Flow cytometry showing increased CD3 + CD8 + T cells in Myeloid-GAS6 KO + α-PD-1. (**C**) Tumor weight reduction in Myeloid-GAS6 KO + α-PD-1. (**D**) Survival curves demonstrating synergy. (**E**) Schematic: GAS6-TYRO3 axis links macrophage metabolism to immunotherapy resistance

To investigate the role of the GAS6-TYRO3 axis in melanoma progression and its impact on immunotherapy efficacy, we generated myeloid-specific GAS6 knockout (Myeloid-GAS6 KO) mice and established a melanoma model by injecting tumor cells into these mice and wild-type (WT) controls. The mice were then treated with anti-PD-1 (α-PD-1) therapy. In WT mice, α-PD-1 treatment showed limited efficacy, whereas in Myeloid-GAS6 KO mice, α-PD-1 treatment significantly enhanced antitumor immunity, as evidenced by reduced expression of immunosuppressive markers (CD163 and TIGIT) and increased expression of the cytotoxic marker perforin in the tumor microenvironment (Fig. 8A). Flow cytometry analysis revealed that Myeloid-GAS6 KO mice exhibited increased infiltration of CD3 + CD8 + T cells even without α-PD-1 treatment, and this effect was further amplified by α-PD-1 therapy compared to WT mice (Fig. 8B). Consistent with these findings, tumor weight measurements showed that Myeloid-GAS6 KO mice had smaller tumors than WT mice, and α-PD-1 treatment further reduced tumor burden, with the most pronounced effect observed in Myeloid-GAS6 KO mice (Fig. 8C). Survival analysis demonstrated that the combination of Myeloid-GAS6 KO and α-PD-1 therapy significantly improved survival rates in melanoma-bearing mice (Fig. 8D).

To assess the potential adverse effects associated with the therapeutic strategies, we systematically evaluated body weight changes, serum biomarkers of organ function, and hematological parameters across all experimental groups. No significant differences in body weight were observed among the groups throughout the treatment period (Supplementary Fig. S3A). Serum biochemical analysis revealed that all key indicators of liver function (ALT, AST), renal function (BUN, creatinine), and pancreatic injury (amylase, lipase) remained within normal ranges across all treatment conditions (Supplementary Fig. S3B). Furthermore, complete blood counts showed no abnormalities in white blood cells, red blood cells, platelets, or hemoglobin levels in any group (Supplementary Fig. S3C). These data indicate that neither myeloid-specific GAS6 deletion nor its combination with α-PD-1 therapy induced detectable systemic toxicity in mice.

Collectively, our results reveal that GAS6 + macrophages enhance glycolysis and oxidative phosphorylation to fuel GAS6 secretion, which interacts with high-risk TEAD3 + melanoma cells via the TYRO3 receptor. This crosstalk dysregulates propionate metabolism in tumor cells, leading to the accumulation of methylmalonic acid and promoting tumor proliferation and aggressiveness. Importantly, targeting the GAS6-TYRO3 axis in Myeloid-GAS6 KO mice sensitizes tumors to α-PD-1 therapy by enhancing CD8 + T cell infiltration and anti-tumor immunity, without inducing observable adverse effects, suggesting a promising and safe strategy to overcome resistance to immune checkpoint blockade in melanoma (Fig. 8E).

## Discussion

In this study, we identified a high-risk subgroup of melanoma characterized by dysregulated short-chain fatty acid (SCFA) metabolism, particularly propionate metabolism pathways, and poor clinical outcomes. Through integrated multi-omics analyses, we uncovered the critical role of TEAD3 in driving melanoma progression and its interaction with GAS6 + macrophages via the GAS6-TYRO3 axis. Furthermore, we demonstrated that myeloid-specific GAS6 knockout enhances the efficacy of anti-PD-1 therapy, providing a novel strategy to overcome immunotherapy resistance in melanoma. Our findings highlight the complex interplay between tumor metabolism, immune modulation, and therapeutic responses, offering new insights into melanoma biology and treatment. TEAD3 emerged as a central regulator of melanoma malignancy, with high expression associated with poor prognosis and aggressive tumor behavior [24, 25]. Our functional validation in vitro and in vivo confirmed that TEAD3 promotes tumor proliferation, invasion, and metastasis by inducing epithelial-mesenchymal transition (EMT). These findings align with previous studies implicating TEAD family transcription factors in cancer progression through their roles in Hippo signaling and metabolic reprogramming [26–28]. The enrichment of TEAD3 in high-risk melanoma cells and its association with M2 macrophage interactions suggest that TEAD3 not only drives intrinsic tumor aggressiveness but also modulates the tumor microenvironment (TME) to favor immune evasion and tumor growth.

Our study revealed that GAS6 + macrophages exhibit a hypermetabolic phenotype, characterized by enhanced oxidative phosphorylation and glycolysis, which fuels GAS6 secretion. This metabolic reprogramming enables GAS6 + macrophages to interact with TEAD3 + melanoma cells via the GAS6-TYRO3 ligand-receptor axis, promoting tumor proliferation, EMT, and metastasis. These findings are consistent with recent reports highlighting the role of GAS6-TYRO3 signaling in cancer progression and immune modulation [29, 30]. The spatial organization of high-risk melanoma cells and GAS6 + macrophages further underscores the importance of spatially defined niches in mediating tumor-immune crosstalk, a concept increasingly recognized in cancer biology [31–33]. We demonstrated that GAS6-TYRO3 signaling dysregulates propionate metabolism in melanoma cells, leading to the accumulation of methylmalonic acid, a metabolite linked to cancer aggressiveness. This metabolic shift was driven by the upregulation of Mmut, which catalyzes the conversion of D-methylmalonyl-CoA to methylmalonic acid. Our findings are supported by recent studies showing that methylmalonic acid accumulation promotes tumor progression by inducing metabolic stress and epigenetic modifications [34, 35]. The functional link between GAS6-TYRO3 signaling and propionate metabolism highlights a novel mechanism by which tumor-immune interactions drive metabolic reprogramming and tumor progression.

One of the most significant findings of this study is the synergistic effect of myeloid-specific GAS6 knockout and anti-PD-1 therapy in enhancing antitumor immunity and improving treatment outcomes. Myeloid-GAS6 KO mice exhibited increased CD8 + T cell infiltration, reduced immunosuppressive markers, and improved survival, even in the absence of anti-PD-1 treatment. When combined with anti-PD-1 therapy, these effects were further amplified, suggesting that targeting the GAS6-TYRO3 axis can sensitize melanoma to immune checkpoint blockade. This is particularly relevant given the limited efficacy of anti-PD-1 therapy in many melanoma patients, which is often attributed to an immunosuppressive TME [36–38]. Our results provide a compelling rationale for combining GAS6-TYRO3 inhibition with existing immunotherapies to overcome resistance and improve patient outcomes. Our study identifies several potential therapeutic targets, including TEAD3, GAS6-TYRO3 signaling, and propionate metabolism. The upregulation of Mmut and the consequent accumulation of methylmalonic acid in high-risk TEAD3 + melanoma cells suggest that targeting these metabolic pathways could disrupt tumor progression mechanisms, particularly in aggressive subtypes such as acral melanoma. Furthermore, the significantly enhanced efficacy of anti-PD-1 therapy in Myeloid-GAS6 KO mice underscores the promising potential of combining GAS6–TYRO3 axis inhibition with immune checkpoint blockade, especially in TEAD3-high acral melanoma—a subtype often associated with innate immunotherapy resistance and poorer clinical outcomes [39–41]. Our findings align with emerging efforts to target immunosuppressive metabolic pathways in cancer [42, 43], and importantly, identify TEAD3 not only as a mediator of aggressiveness but also as a potential biomarker for stratifying patients who may benefit from combined metabolic-immune targeting.

While this study provides mechanistic insight into melanoma progression and immunotherapy resistance, certain limitations must be acknowledged. First, our experimental models primarily reflect features of acral melanoma and the identified high-risk TEAD3 + subgroup; thus, the generalizability to other melanoma subtypes (e.g., BRAF or NRAS mutant, or triple wild-type tumors) requires further validation. Although we have supplemented our in vitro studies with additional cell lines (A375, MM9H-1), future research should include more diverse genetic backgrounds and patient-derived xenograft models to evaluate the broader applicability of our findings. Second, while our toxicity assessments indicated no significant adverse effects from myeloid-specific GAS6 deletion combined with anti-PD-1 in mice—with no marked changes in body weight, serum organ function markers, or hematological parameters (Supplementary Fig. S3)—the translational relevance and safety profile in humans must be confirmed through clinical trials. In particular, TYRO3 inhibition may pose theoretical risks such as retinal dysfunction or altered platelet activity [44–47], and combination immunotherapy could exacerbate immune-related adverse events; these possibilities warrant careful monitoring in future studies. Finally, the precise mechanisms through which methylmalonic acid promotes tumor aggressiveness and modulates CD8⁺ T cell function demand further in-depth investigation.

In conclusion, our study elucidates the pivotal role of the GAS6–TYRO3 axis and TEAD3-driven metabolic reprogramming in promoting melanoma progression and immune evasion, with particular relevance in acral and other high-risk melanomas. By integrating multi-omics and functional validation, we provide a rationale for stratifying patients based on TEAD3 expression and propose targeting GAS6 signaling or methylmalonic acid accumulation as a novel combinatorial strategy with immune checkpoint inhibitors. These insights reinforce the importance of understanding tumor-immune-metabolic crosstalk within the tumor microenvironment and highlight the potential of biomarker-guided therapy in melanoma.

## Supplementary Information

Below is the link to the electronic supplementary material.

Supplementary Material 1

## Data Availability

Data are available upon reasonable request.

## Abbreviations

CAFs: Cancer–associated fibroblasts
CCK-8: Cell counting kit–8
2-DG: 2–Deoxy–D–Glucose
ECAR: Extracellular acidification rate
EGF: Epidermal growth factor
EMT: Epithelial–mesenchymal transition
GAS6: Growth arrest–specific 6
GEO: Gene expression omnibus
GLTP: Glycolipid transfer protein
GO: Gene ontology
GSEA: Gene set enrichment analysis
KEGG: Kyoto encyclopedia of genes and genomes
Mmut: Methylmalonyl–CoA mutase
OCR: Oxygen consumption rate
OXPHOS: Oxidative phosphorylation
PD-1: Programmed cell death protein 1
TAMs: Tumor–associated macrophages
TCGA: The cancer genome atlas
TEAD3: Transcriptional enhanced associate domain 3
TGF-β: Transforming growth factor beta
TIGIT: T Cell Immunoreceptor With Ig And ITIM Domains
TME: Tumor microenvironment
TYRO3: TYRO3 protein tyrosine kinase
VISTA: V–Domain Ig suppressor of T cell activation
