# Supplementary material for: TEAD3 + high-risk melanoma cells crosstalk with GAS6 + macrophages via the GAS6-TYRO3 ligand-receptor axis to modulate propionate metabolism and drive melanoma progression
Source: J Exp Clin Cancer Res. 2025 Oct 1;44:279. doi: 10.1186/s13046-025-03542-0 (PMC12486507; doi:10.1186/s13046-025-03542-0)
Supplement: Supplementary file 1 — Supplementary Material 1 [file 13046_2025_3542_MOESM1_ESM.pdf]

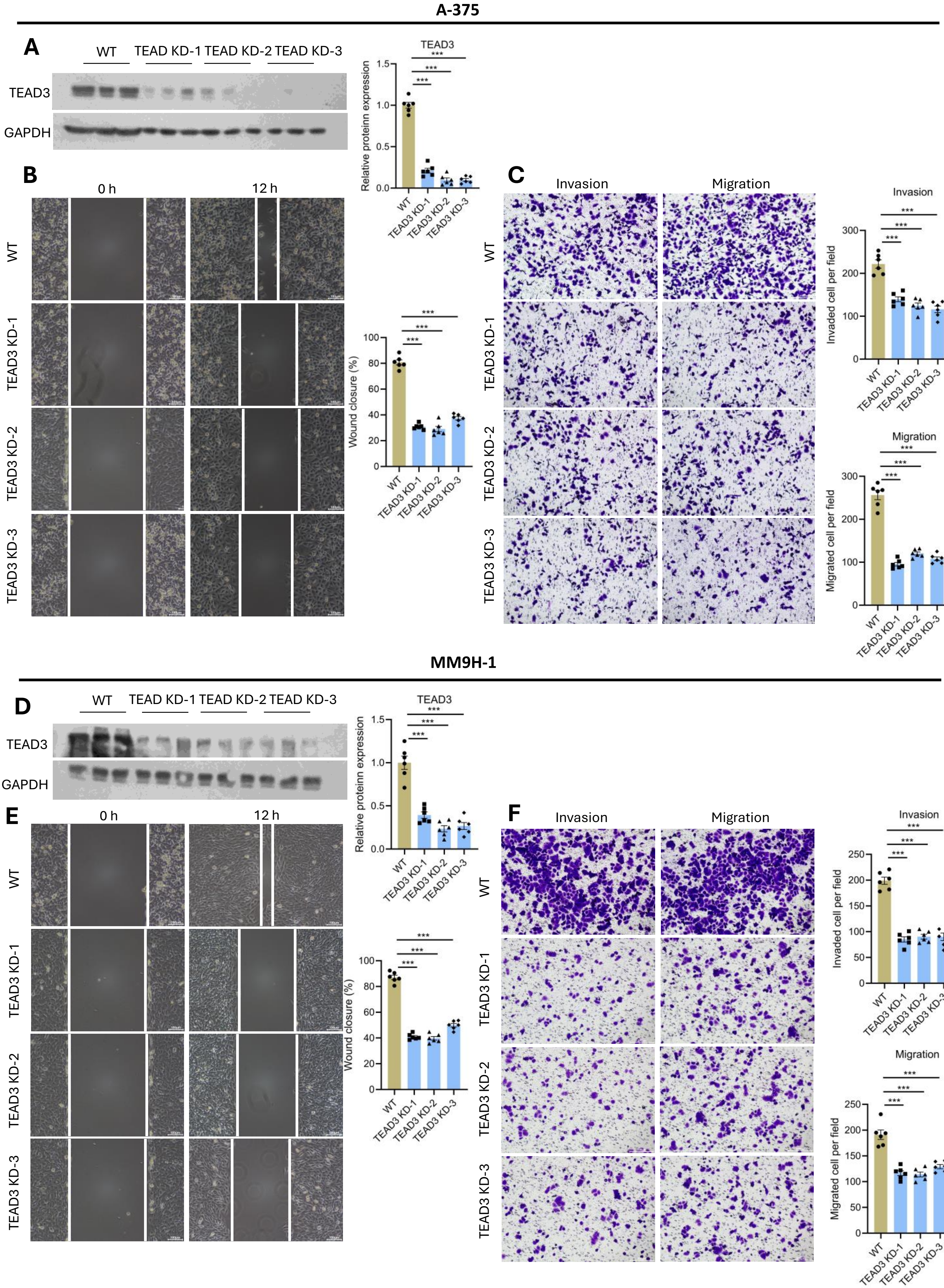

Supplementary figure S1

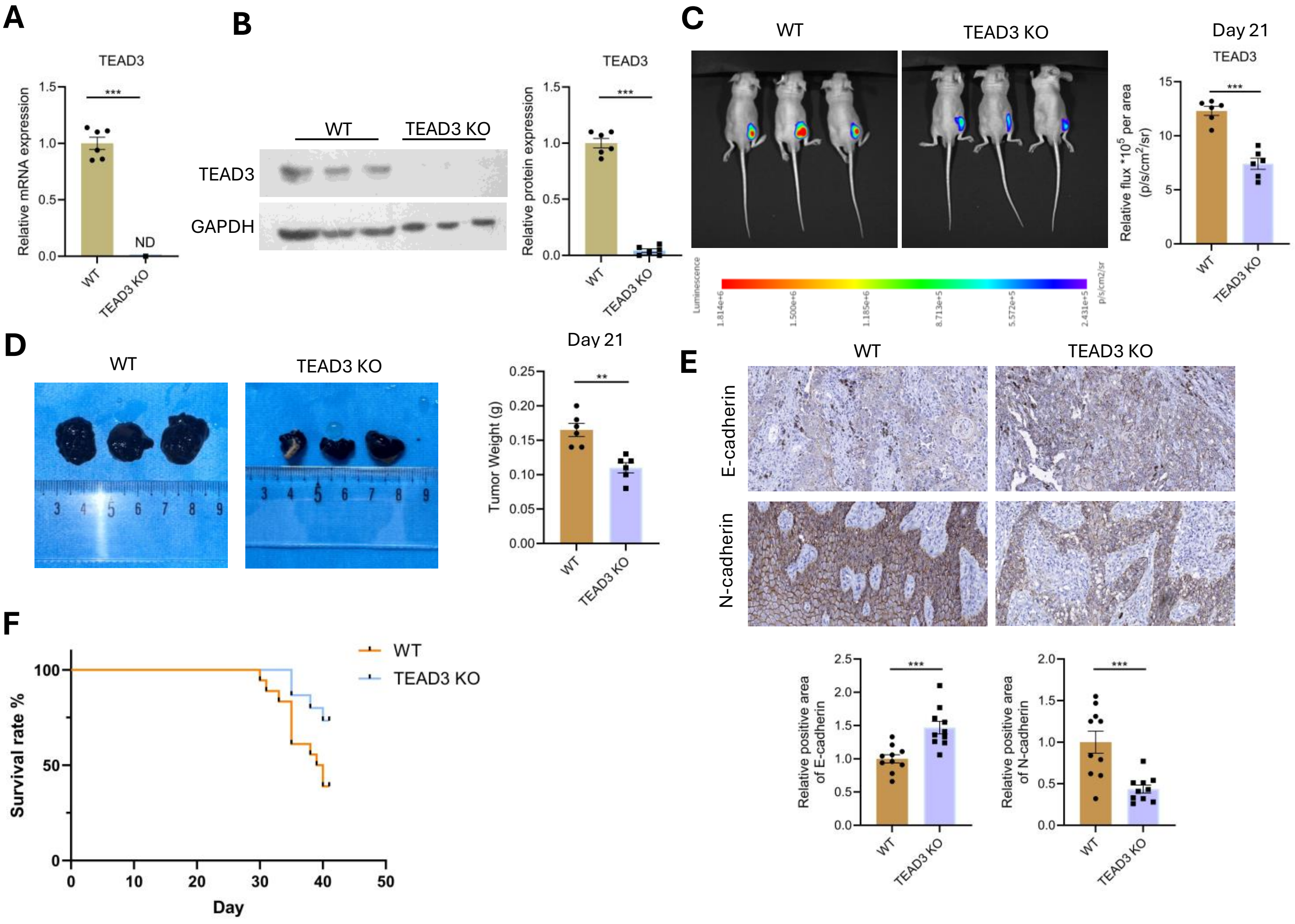

Supplementary figure S2

		PBS (control)	Anti-PD-1 monotherapy	Myeloid-specific GAS6 knockout (PBS-treated)	Myeloid-specific GAS6 knockout combined with anti-PD-1
Liver function	ALT	32.5±4.2 U/L	35.1±3.8 U/L	30.8±3.5 U/L	33.6±4.1 U/L
	AST	58.3±6.2 U/L	61.0±5.7 U/L	56.5±4.9 U/L	59.4±6.5 U/L)
Renal function	BUN	18.2±2.3 mg/dL	19.5±2.6 mg/dL	17.8±2.1 mg/Dl	18.9±2.4 mg/dL
	Creatinine	0.41±0.05 mg/dL	0.43±0.06 mg/dL	0.40±0.04 mg/dL	0.42±0.05 mg/dL
Pancreatic injury	Amylase	812±98 U/L	835±105 U/L	798±92 U/L	820±101 U/L
	lipase	52.3±6.4 U/L	54.1±7.0 U/L	50.8±6.1 U/L	53.2±6.7 U/L
Hematological profiles	WBC (×10³/μL)	6.8±1.2	7.2±1.7	6.6±1.1	7.0±0.9
	RBC (×10^6/μL)	8.5±0.6	8.7±0.5	8.4±0.6	8.6±0.5
	Platelets	985±112	1012±120	974±108	996±115
	Hemoglobin	13.8±1.4	14.0±1.1	13.7±1.2	13.9±1.0

Supplementary figure S3
